# Clinical presentation, auscultation recordings, ultrasonographic findings and treatment response of 12 adult cattle with chronic suppurative pneumonia: case study

**DOI:** 10.1186/2046-0481-66-5

**Published:** 2013-04-01

**Authors:** Philip R Scott

**Affiliations:** 1 Easter Bush Veterinary Centre, Roslin, Midlothian, Scotland, EH25 9RG, UK

**Keywords:** Ultrasonography, Respiratory disease, Cattle, Auscultation, Diagnosis, Trestment

## Abstract

Auscultation is considered the critical component of the veterinary clinical examination for the diagnosis of bovine respiratory disease but the accuracy with which adventitious sounds reflect underlying lung pathology remains largely unproven. Modern portable ultrasound machines provide the veterinary practitioner with an inexpensive, non-invasive tool with which to examine the pleural surfaces and superficial lung parenchyma. Simultaneous recording of sounds overlying normal lung and defined pathology allows critical assessment of auscultated sounds in the same animal removing confounding factors such as respiratory rate and thickness of the chest wall (body condition). Twelve cows, referred to the University of Edinburgh Veterinary School, were diagnosed with chronic suppurative pneumonia and enrolled into this prospective study to record and monitor lung sounds, ultrasonographic findings, and response to a standardised antibiotic treatment regimen.

Most cows (8/12) had a normal rectal temperature on presentation but all cows had received antibiotic therapy at some time in the previous two weeks and six animals were receiving antibiotic treatment upon admission. All cattle were tachypnoeic (>40 breaths per minute) with frequent and productive coughing, halitosis, and a purulent nasal discharge most noticeable when the head was lowered. Ultrasonographic examination of the chest readily identified pathological changes consistent with severe lung pathology subsequently confirmed as chronic suppurative pneumonia in four cows at necropsy; eight cows recovered well after antibiotic treatment and were discharged two to six weeks after admission. It proved difficult to differentiate increased audibility of normal lung sounds due to tachypnoea from wheezes; coarse crackles were not commonly heard. In general, sounds were reduced in volume over consolidated lung relative to normal lung tissue situated dorsally. Rumen contraction sounds were commonly transmitted over areas of lung pathology.

*Trueperella (formerly Arcanobacterium) pyogenes* was isolated from three of four lung tissue samples at necrospy. Treatment with procaine penicillin for 42 consecutive days resulted in marked improvement with return to normal appetite and improvement in body condition in 8 of 12 cows (67%) where lesions did not extend more than 10-15 cm above the level of the olecranon on both sides of the chest.

## Background

**Figure 1:**
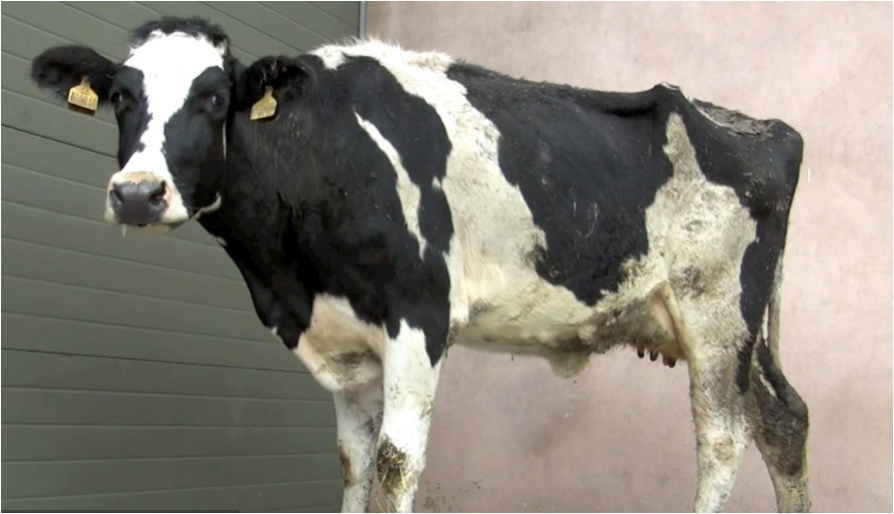
**Typical appearance of recently calved Holstein heifer at presentation.** All cattle were dull and depressed with poor rumen fill.

**Figure 2:**
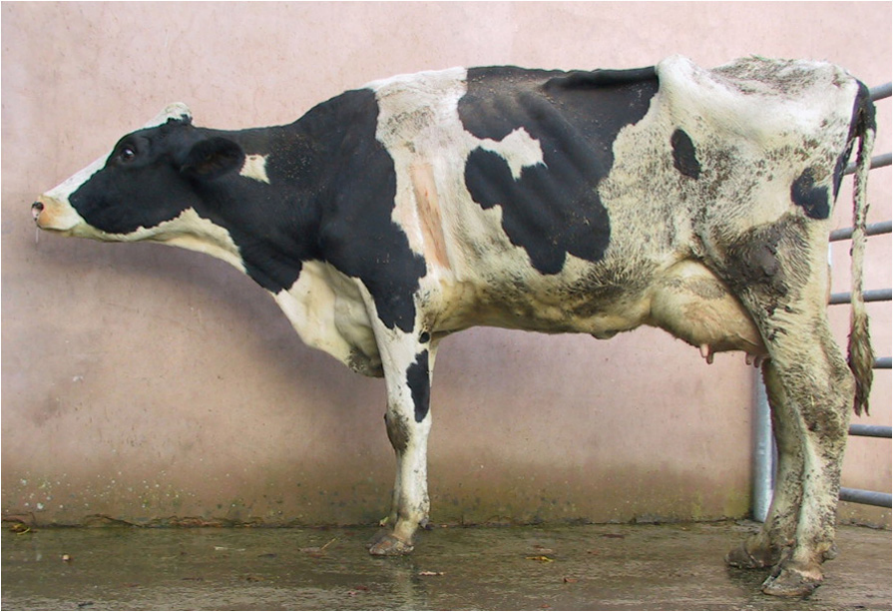
Cattle with chronic suppurative pneumonia typically stood with the neck extended and the head held lowered.

**Figure 3:**
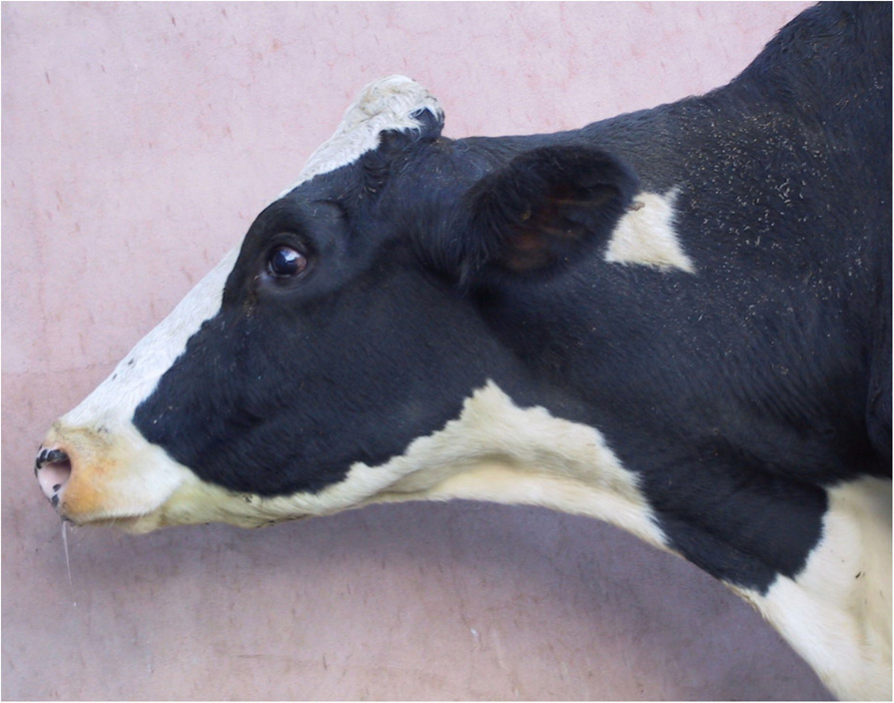
Subjective visual appraisal suggested that cattle with chronic suppurative pneumonia had a painful expression.

**Figure 4:**
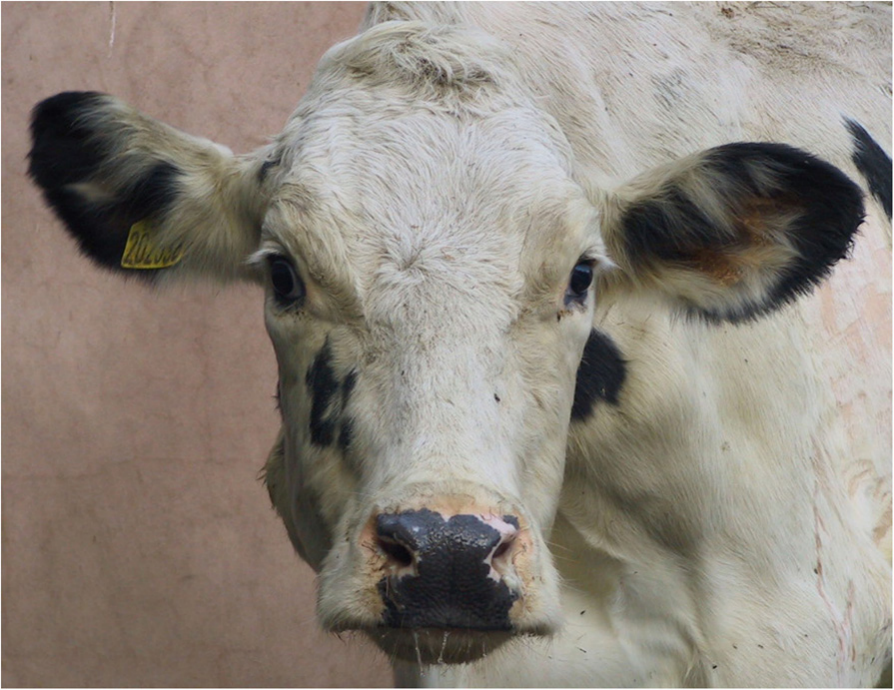
A purulent nasal discharge was present intermittently in cattle with chronic suppurative pneumonia.

Chronic suppurative pneumonia is a difficult diagnosis in farm animal practice Figures 1, 2 and 3 because cattle have often been treated by the farmer before presentation, and other chronic bacterial infections such as peritonitis, endocarditis, pericarditis, liver abscessation, pyelonephritis, and metritis can be presented with poor production and weight loss. Cows affected by chronic suppurative pneumonia are commonly afebrile at presentation even when there has been no prior antibiotic administration. Purulent nasal discharge Figure 4 and frequent coughing are common presenting signs in cattle with chronic suppurative pneumonia but these signs are also present in infectious bovine rhinotracheitis, a disease where there are no lung lesions.

Descriptions of adventitious sounds are very variable and the extent to which these sounds can be auscultated over specific lung pathologies has been questioned in ovine respiratory diseases [1-3]. The ability to ascertain the nature and distribution of lung pathology by auscultation remains unproven [4] despite the fact that it remains the cornerstone of clinical examination of the ruminant respiratory tract [5,6].

Reference textbooks on clinical examination describe abnormal lower respiratory sounds in ruminants as clicking, popping or bubbling sounds, crackling sounds, wheezes, and pleuritic friction rubs [7]. A wide range of descriptors has also been used in the clinical literature for abnormal lung sounds in sheep including increased vesicular sounds for a ram with severe chronic suppurative pleuropneumonia [8], and wheezing, rubbing vesicular and murmuring sounds in sheep with bacterial respiratory infections, followed by absence of residual bronchial catarrh in the same sheep during recovery [9]. However, authors in more recent papers [10,11] have limited their descriptions of auscultation findings of the respiratory tract to distribution rather than character; no abnormal sounds recorded (score 0), abnormal sounds audible predominantly anteroventrally (score 1), abnormal sounds audible throughout the entire lung field (score 2), or have simply commented in a more general sense on the presence of ‘loud and prolonged respiratory sounds’ [12]. The lack of correlation between lungs sounds and distribution of pathology in “wheel-barrow negative” ovine pulmonary adenocarcinoma (OPA) cases [4], despite the OPA lesions extending to involve up to 20% of lung tissue, serves to emphasise the apparent lack of sensitivity and specificity of auscultation.

The purpose of this article is to present images and video recordings highlighting the clinical, sonographic, and necropsy findings of cows with chronic suppurative pnemonia. Sounds recorded over typical ultrasound images are presented with the lesion type confirmed at necropsy in those cattle that did not respond to antibiotic treatment.

Alternative approaches to the diagnosis of chronic suppurative pneumonia in general practice include the response to antibiotic therapy although this regimen would also treat chronic bacterial infections affecting other organs and does not confirm the presence of lung pathology. Radiography could be attempted but presents practical problems with access to the cranio-ventral lung lobes, health and safety regulations, cost, and availability of suitable x-ray machines. Isolation of recognised respiratory tract pathogens from the lower airways and lung via broncho-alveolar lavage and trans-tracheal wash indicates infection but does not quantify the extent of the disease process.

This case study presents clinical findings, including sound recordings over normal and diseased lung and ultrasound images, from adult cattle with chronic suppurative pneumonia. Interpretation of auscultated sounds presents numerous problems and may often be mistakenly interpreted that there is little or no lung pathology present. The primary aim of this report is to demonstrate to veterinary colleagues that ultrasonography is a cost-effective and practical ancillary test in the investigation of suspected respiratory disease on farm. Encouraging results have been achieved in some cases using an extended course of procaine penicillin injections where previous antibiotic therapy, primarily with fluoroquinolones, had failed to effect any improvement. Veterinarians are coming under increasing pressure to reduce the amounts of antibiotics they prescribe because of the perceived association between antibiotic use in farmed species and the rise in multiple antibiotic resistant bacteria in humans. This study provides evidence that extended duration of penicillin therapy achieves good success in selected cases of chronic suppurative pneumonia; veterinarians should combine data from their daily clinical work to provide the necessary information to maintain their right to prescribe.

### Case description

#### Cases

The Farm Animal Hospital at the University of Edinburgh receives ruminant cases from local veterinary practices in south-east Scotland. Twelve adult cattle with chronic suppurative pneumonia admitted over a two year period (2009–2011) were examined by the author and included in this study; 11 Holstein dairy cattle including seven recently-calved heifers, and one pedigree Simmental cow. Adult cattle with other causes of lung pathology were excluded from the study. Six animals were receiving treatment at admission comprising a fluoroquinolone antibiotic (five cows) and oxytetracycline (one cow).

#### Clinical examination findings

The duration of clinical signs was reported to be between 5–14 days although this could not be verified. All cows were in poor body condition (median 1.5; range 1.5-2.5, scale 1–5). Appetite was markedly reduced and all animals refused their concentrate ration. Milk production was reported to be 25 to 50 per cent of expected yield. Eight cows, including four receiving antibiotics, presented with a sub-normal to normal rectal temperature (37°C to 38.5°C); four cows showed a slight fever (39°C to 39.2°C) including two cows receiving antibiotic treatment. All cows were reluctant to move, dull and depressed and typically stood with the neck extended and the head held lowered. Two cows were dyspnoeic at presentation. Subjective visual appraisal suggested that all cows had an anxious/painful expression. All cows coughed repeatedly during the clinical examination, ultrasonographic and sound recording session which lasted for a total of approximately 15 minutes. A purulent unilateral or bilateral nasal discharge was present intermittently but was noted in all cows at some stage during the examination period. The respiratory rate was increased in all animals above 40 breaths per minute. Marked flaring of the nostrils was observed during inspiration in four animals. No other significant infections were detected during the clinical examination or at necropsy of four cows which did not respond to treatment. Cows were treated with 12 mg/kg procaine benzylpenicillin injected intramuscularly once daily into the muscles of the neck or hindquarters. Four cows discharged before the end of the 42 days’ treatment course (two cows after 14 days; two cows after 30 days) were treated by the owner. The importance of completing the treatment course was emphasised to the owner and referring veterinarian but compliance could not be guaranteed.

## Methods

### Ultrasonographic examination

A 5.0 MHz sector transducer connected to a real-time, B-mode ultrasound machine provided diagnostic quality images in all 12 cattle. A 7 to 10 cm wide strip of hair was shaved from both sides of the thorax extending in a vertical plane from the point of the elbow to the caudal edge of the scapula. The skin was soaked with warm tap water then ultrasound gel liberally applied to the wet skin to ensure good contact. The transducer head was firmly held at right angles against the skin overlying the intercostal muscles of the 6th or 7th intercostal spaces. The dorsal lung field was selected at the start of all ultrasound examinations to visualize normal lung tissue as this area is much less commonly affected in chronic suppurative pneumonia The ultrasonographic examinations were made with an initial depth setting of 8 to 10 cm including 2.5 cm of chest wall. Good contact between the transducer head and skin overlying an intercostal space was evident by the breadth and intensity of the ultrasound image.

It was important to visualise the echogenic (white) line of the normal visceral pleura [13] before scanning the more ventral areas. Artefacts can readily be created if the field includes the shoulder musculature. If there was doubt concerning the imaged lesion the visceral pleura was followed down the chest wall to identify the junction between normal lung and pathology.

### Sound recordings

Sound recordings were made using a standard stethoscope head connected to a microphone (Olympus ME-15; Misco) by a short piece of plastic tubing. The electrical output of the microphone was pre-amplified before digitization and storage using a commercial voice recorder (Olympus WS-321M; Misco). Sound files were saved as .wav files. Sensitivity was maintained at the same level for all recordings permitting audibility comparison between recordings. Each recording lasted 60 s and was repeated if there was significant animal movement or other interruption. All efforts were made to exclude extraneous noise. All recordings were undertaken with the cow standing with minimal restraint.

Sound recordings were taken from all cows upon admission and from 8 cows at two weekly intervals until discharge from the hospital 14 to 42 days later.

## Results

### Ultrasonographic findings

#### Normal cattle

The lungs of 30 adult cattle, euthanased for reasons other than respiratory disease, were scanned before necropsy to characterise the sonographic appearance of normal lung tissue. Normal lung was characterized by the uppermost white linear echo with equally-spaced reverberation artefacts below this line. In normal adult cattle (around 600 kg) the visceral pleura was observed moving approximately 5 mm in a vertical plane during respiration. No pleural fluid was visualized in these normal cattle. The chest wall was approximately 2 to 3 cm wide.

#### Chronic suppurative pneumonia

**Figure 5:**
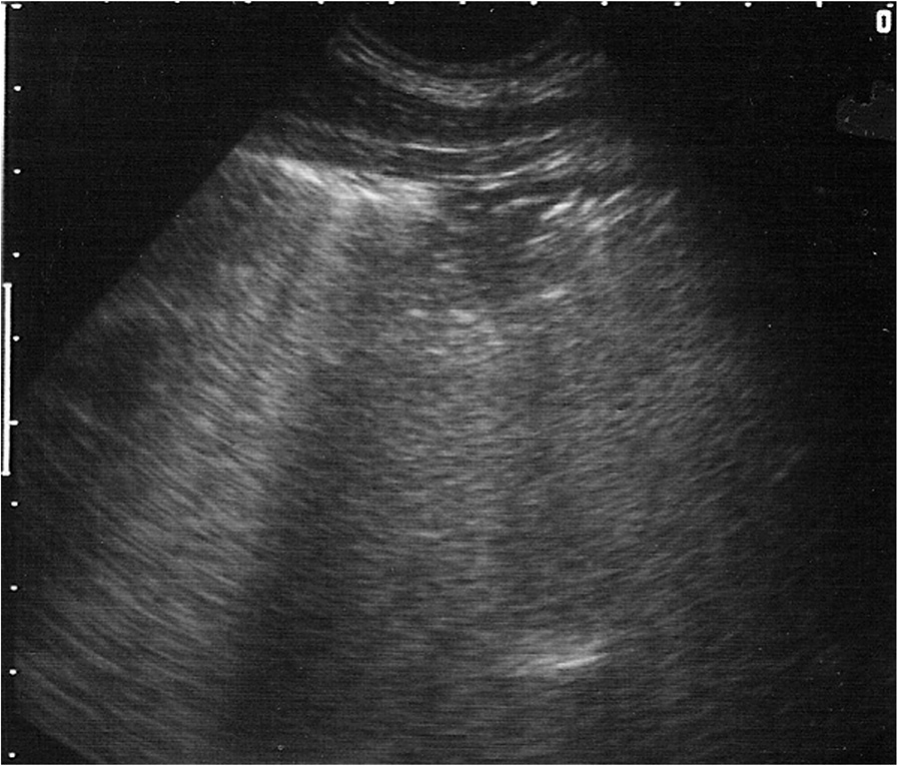
**Ultrasound image generated using a 5 MHz sector scanner.** The probe head is at the top of the image; dorsal is to the left. The hyperechoic (white) line representing the normal visceral pleura (lung surface) extends from the left hand margin half way across the image where it is replaced by a 6 cm deep hypoechoic area which represents cellular infiltration of a lung lobule.

**Figure 6:**
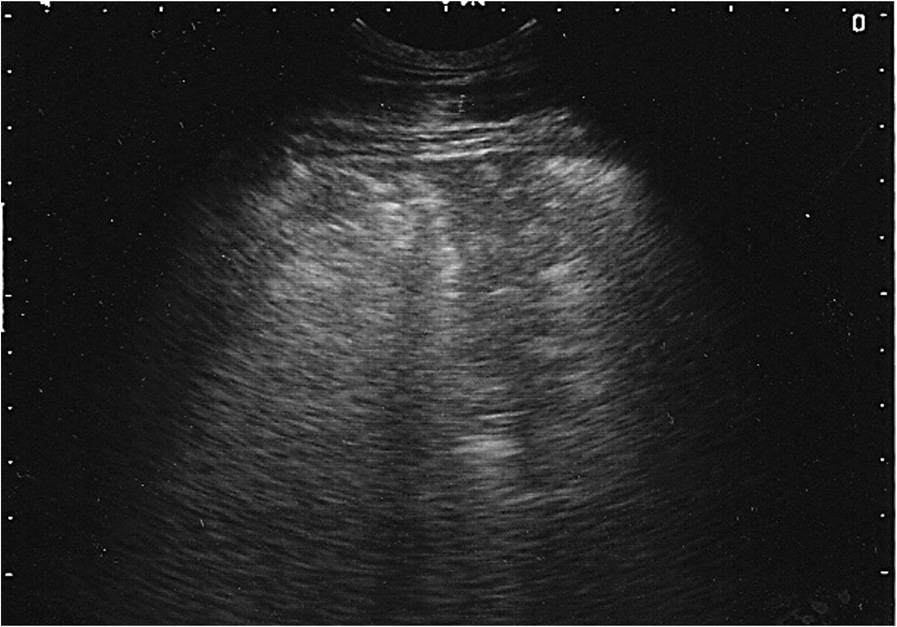
**Ultrasound image generated using a 5 MHz sector scanner.** The probe head is at the top of the image; dorsal is to the left. The hyperechoic (white) line representing the normal visceral pleura (lung surface) extends from the left hand margin half way across the image where it is replaced by a 7 cm deep hypoechoic area which represents cellular infiltration of a lung lobule.

**Figure 7:**
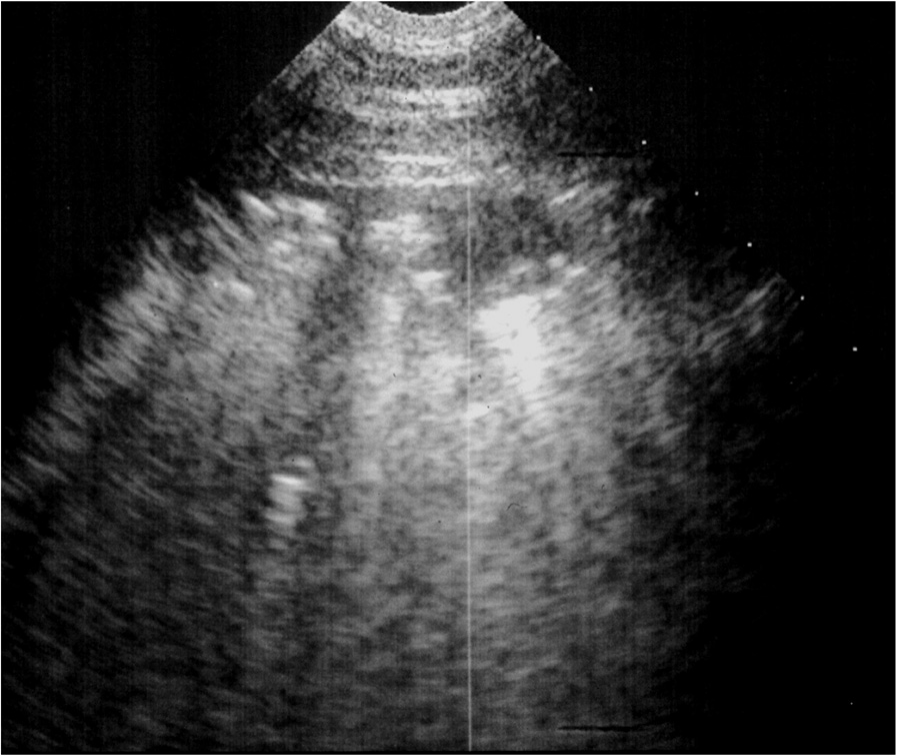
**Ultrasound image generated using a 5 MHz sector scanner.** The probe head is at the top of the image; dorsal is to the left. The hypoechoic columnar irregularity interrupting the continuous white linear echo of the normal lung surface represents consolidated lung lobules.

**Figure 8:**
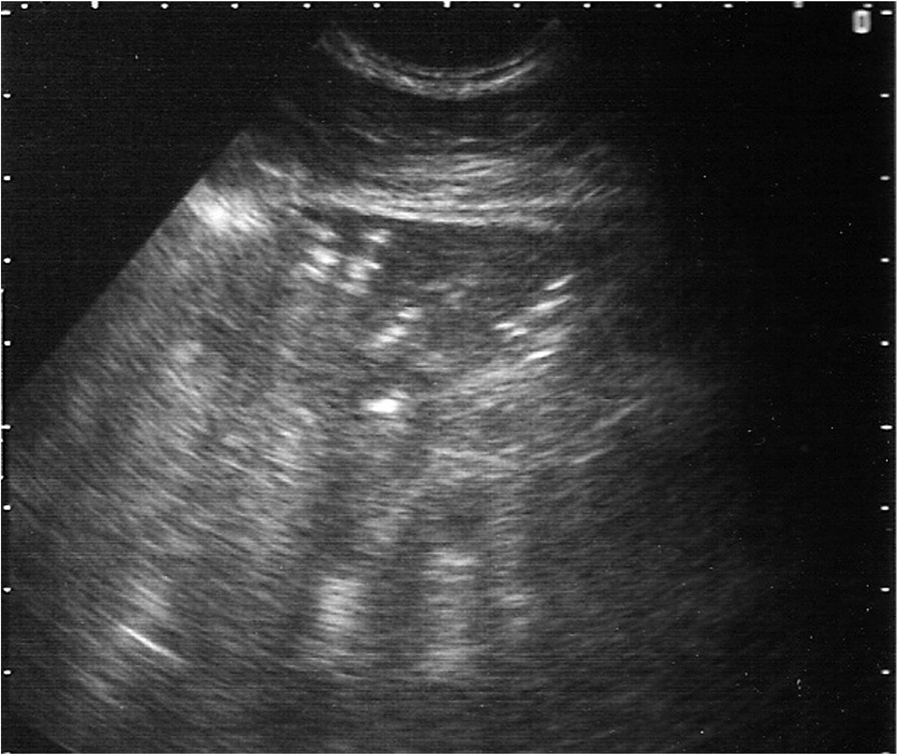
**Ultrasound image generated using a 5 MHz sector scanner.** The probe head is at the top of the image; dorsal is to the left. The hypoechoic columnar irregularity replacing the white linear echo of the normal lung surface represents consolidated lung lobule.

**Figure 9:**
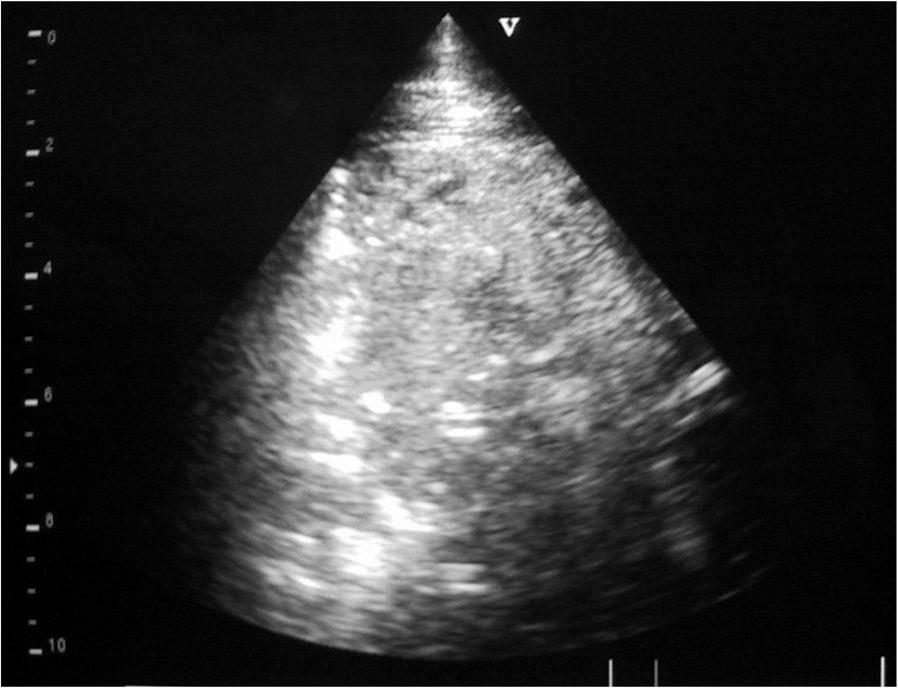
**Ultrasound image generated using a 5 MHz sector scanner (case 1).** The probe head is at the top of the image; dorsal is to the left. The hypoechoic area extending for 8 cm in the ventral lung field represents consolidated lung.

**Figure 10:**
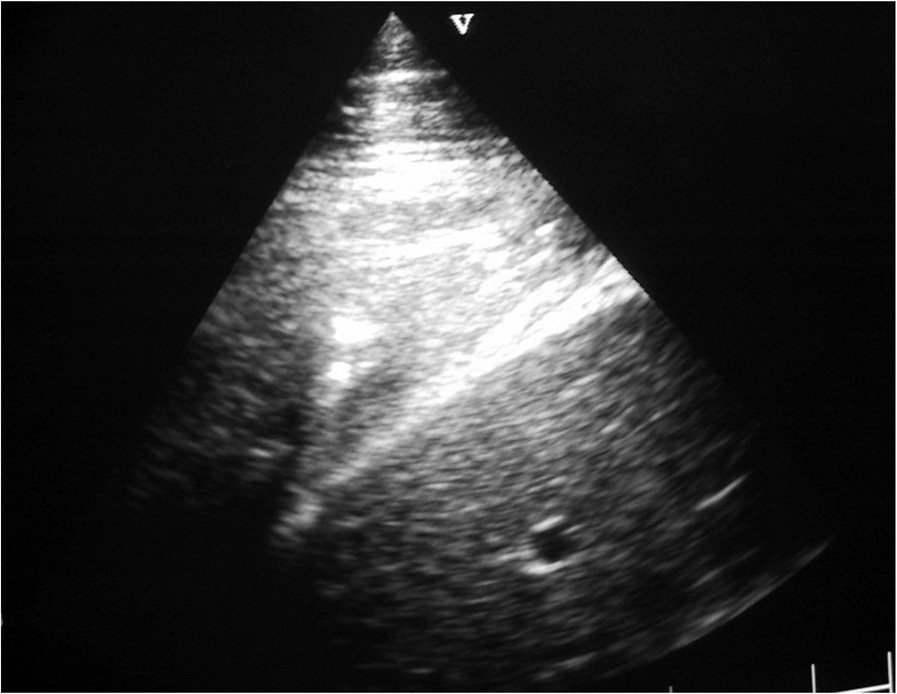
**Ultrasound image generated using a 5 MHz sector scanner (case 3).** The probe head is at the top of the image; dorsal is to the left. The hypoechoic lung transmits sound waves through the diaphragm to image the liver. The consolidated lung featured has the sonographic appearance of liver.

As the probe head was moved ventrally there was an abrupt change from normal lung, represented by the bright hyperechoic visceral pleura, to be replaced by hypoechoic areas measuring approximately 2 cm in the vertical plane and extending for 2–6 cm into the lung parenchyma in 8 of 12 cattle Figures 5 and 6. These columns resulted from increased sound wave transmission through consolidated lung parenchyma, typically in a lobular pattern, then striking normal lung tissue/small airways. Ventrally, these hypoechoic columns Figure 7 merged to form a large hypoechoic area with the sonographic appearance of liver extending up to 10 cm from the viscera pleura containing many hyperechoic lines measuring up to 2 to 10 mm Figures 8, 9 and 10. In four cows, there was a sudden change from normal lung to consolidated lung parenchyma without the lobular consolidation pattern described above.

**Figure 11:**
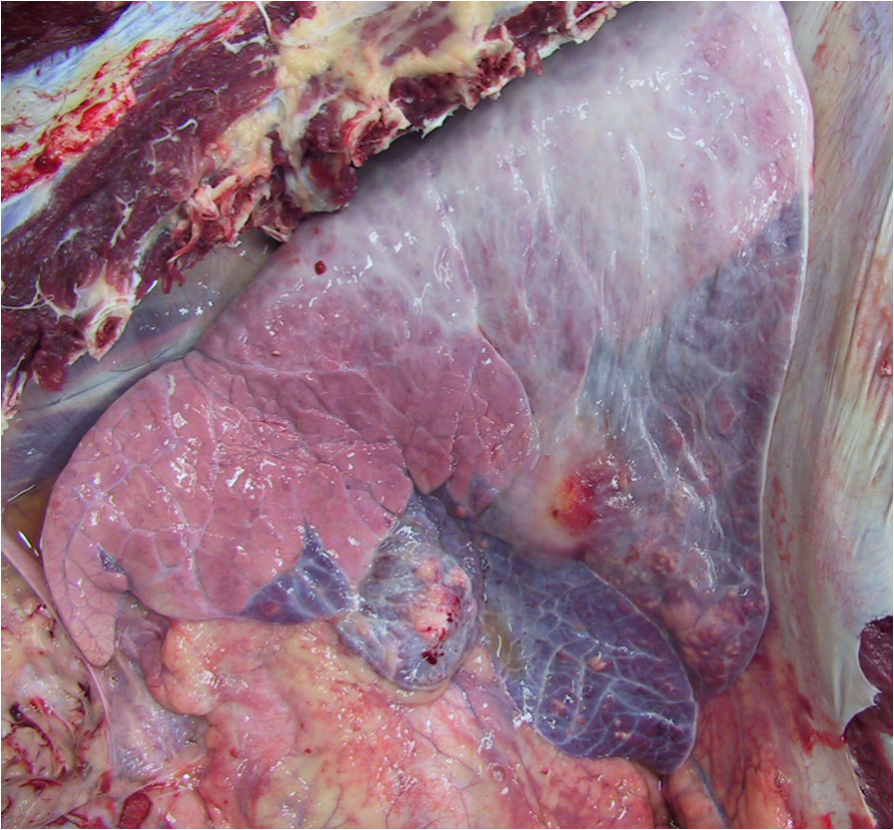
**Necropsy revealing sharply-demarcated lung pathology in the ventral portions of the lung lobes of a cow with chronic suppurative pneumonia that did not respond to antibiotic therapy.** The pathology affects approximately 30 per cent of the right lung in this animal.

**Figure 12:**
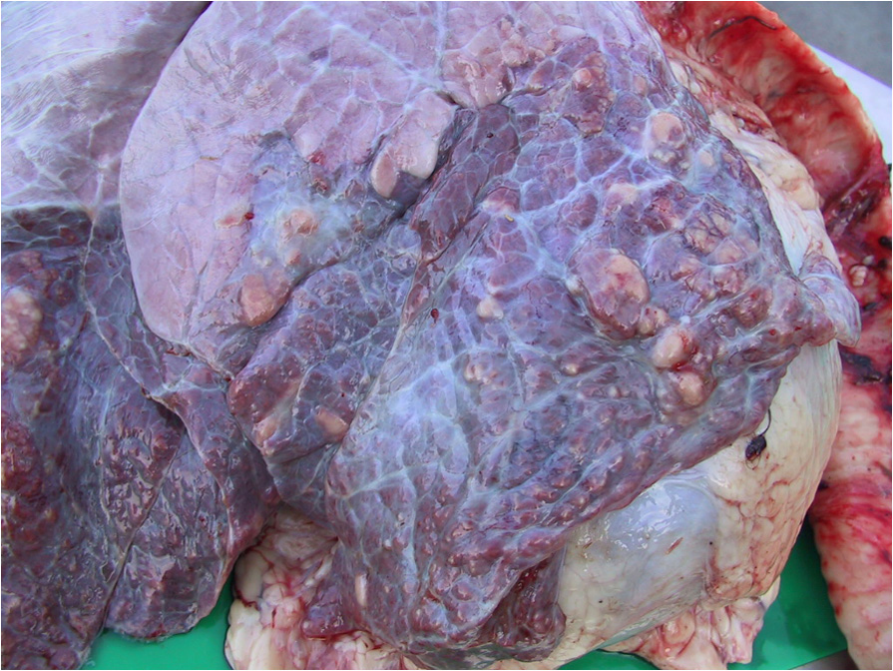
**Necropsy revealing the pluck of a cow with chronic suppurative pneumonia that did not respond to antibiotic therapy.** There is a sharp demarcation between normal lung and lung consolidation in the ventral portions of the lung lobes.

**Figure 13:**
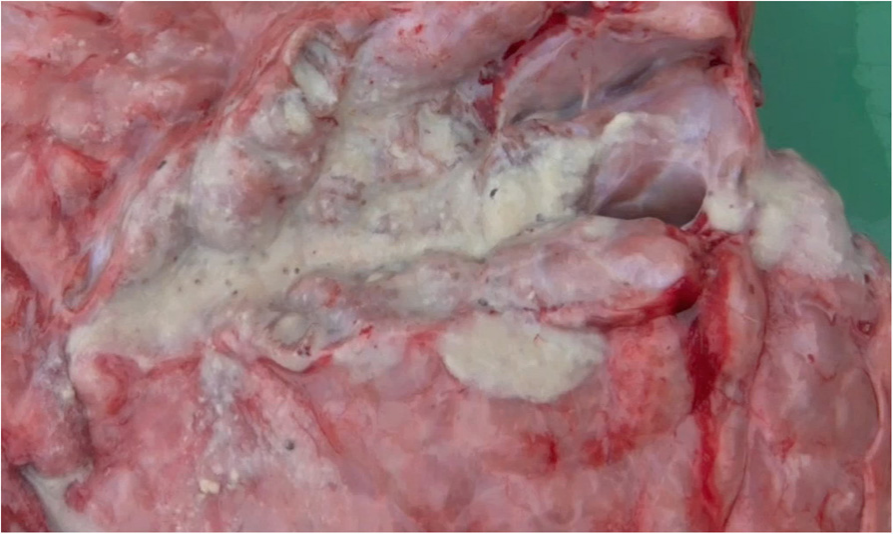
**Cut section at necropsy through consolidated lung represented in Figure**9.

**Figure 14:**
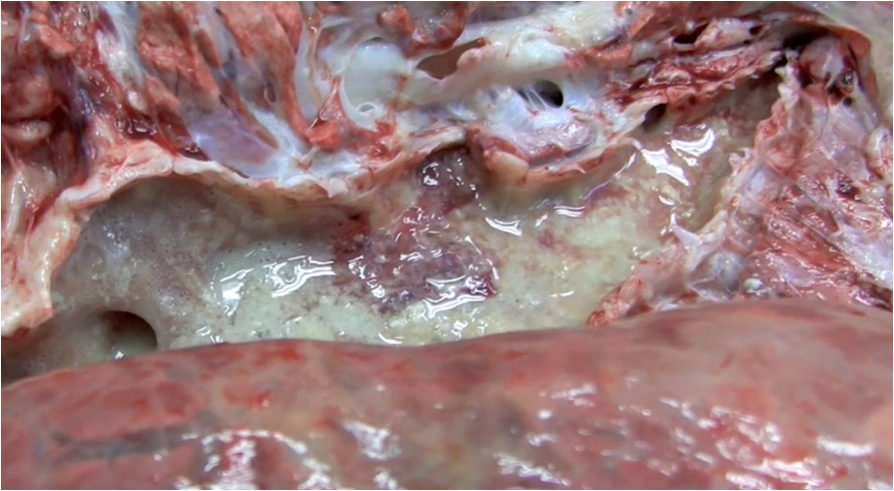
**Cut section through major airways revealing large quantities of purulent material (see also Figures**9**and**13**from same case).**

Few discrete lung abscesses were observed at necropsy and the hypoechoic areas identified during ultrasonographic examination represented consolidated lung parenchyma Figures 11 and 12, with a distinct lobular appearance dorsally, with purulent material within smaller airways (bronchiectasis) Figures 13 and 14. *Trueperella (Arcanobacterium) pyogenes* was the most common bacterium isolated from diseased lungs (3 out of 4 cows sampled at necrospy).

#### Sound recordings

It proved difficult to differentiate increased audibility of normal breath sounds from wheezes. When wheezes were identified, they were audible over the whole lung field and not restricted to the areas of lung pathology identified during ultrasound examination. In some cases, wheezes were reduced over lung pathology. Few crackles could be identified over lung pathology. Crackles were not consistently heard in four cows despite the presence of purulent material expressed from the airways of affected lung at necropsy. Transmitted gut sounds were often heard over consolidated lung Additional files 1 and 2 containing six and three sound recordings respectively.

### Analyses

#### Ultrasonographic diagnosis

Ultrasonographic examination accurately defined the distribution of lung pathology revealed at necropsy in the four cows that did not respond to antibiotic therapy. The sonographic appearance of lung lesions recorded from these four cows was similar to the eight cows successfully treated although the distribution, as defined by the dorsal margin, was considerably more extensive. The sonographic changes on both sides of the thorax totalled more than 40 cm above the level of the olecranon in the four cows that did not respond to antibiotic therapy and were euthanased for welfare reasons. The area involved in the disease process was estimated to represent more than 30 per cent of lung surface area. The lung changes could not be confirmed in the eight cattle discharged from the hospital.

#### Treatment efficacy

**Figure 15:**
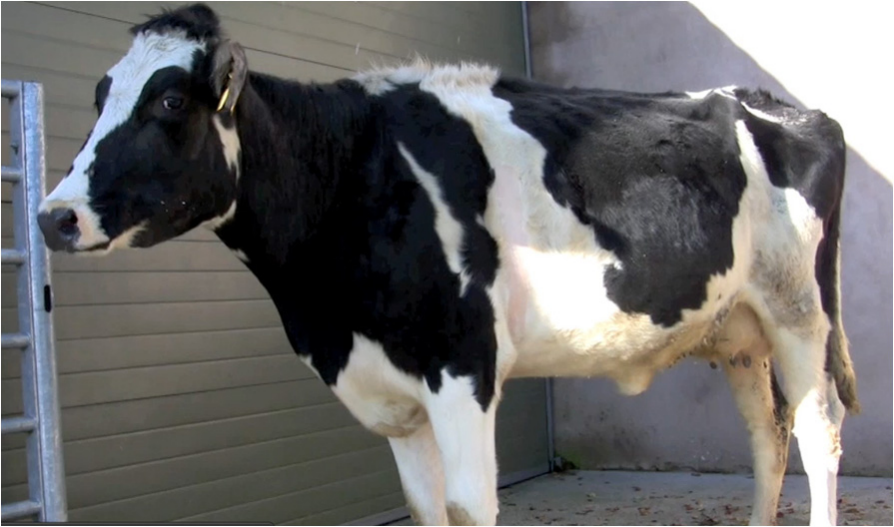
**Cow diagnosed with chronic suppurative pulmonary disease (Figure**1**) after 10 consecutive days’ treatment with penicillin (see above).** The animal appears more alert, there is no nasal discharge, and the left flank is not sunken suggestive of normal rumen fill.

Rectal temperature was normal within 48 hours of treatment in the four cows that had a slight fever at presentation. The clinical appearance and appetite were improved in two cows, much improved in eight cows within 48 hours but did not improve in two cows which were dyspnoeic at presentation. Milk yield improved in two cows but six cows were dried off prematurely for management reasons. Coughing and purulent nasal discharges were much reduced after one week. There was no localised reaction to repeated intramuscular penicillin injections. Eight cows were discharged after two to six weeks Figure 15. Four cows were sold for slaughter two to three months after discharge having gained body condition; four cows remained in the herd.

## Discussion and evaluation

### Diagnosis

Clinical findings of reduced appetite, poor production, coughing, purulent nasal discharge and tachypnoea in all cases were consistent with previous reports of chronic suppurative pneumonia [14,15]. Pyrexia was not a feature of chronic suppurative pneumonia in this study albeit all cows had received antibiotics at some stage during the previous two weeks however these antibiotic treatments had not effected any clinical improvement. This situation differs significantly from acute respiratory disease in growing cattle where fever >39.7°C is considered the most important selection criterion for antibiotic therapy [16,17].

In the present study it proved difficult to differentiate increased audibility of normal breath sounds from wheezes; no crackles were detected over lung pathology identified ultrasonographically. In most cases, breath sounds were markedly reduced over the lung pathology. The absence of adventitious sounds, particularly crackles, is explained by the lack of air movement caused by extensive lung consolidation in these areas which had the sonographic appearance of liver. The accuracy of the sonographic findings were confirmed in all four cases at necropsy. When wheezes were identified, they were audible over the whole lung field and not restricted to the areas of lung pathology identified during ultrasound examination.

While abnormal auscultatory findings have been described over the cranioventral lung field of cattle with chronic suppurative pneumonia no details were supplied [18]. Progression of the disease process to the stage that severe respiratory disease exists is defined whereby expiratory sounds are as harsh as inspiratory sounds [19] did not identify severely affected cattle in the present study.

Written descriptions of adventitious lung sounds are inadequate. Veterinary clinicians are encouraged to decide for themselves whether auscultation is useful in reaching the specific diagnosis of chronic suppurative pneumonia by examining the sound recordings in this report from normal lung and over specific pathology determined ultrasonographically. Sound recordings over specific ovine lung pathology are available to download for comparison [1,3].

Few crackles could be identified over lung pathology despite the presence of purulent material that could be expressed from the airways of affected lung at necropsy. No readily discernible differences in lungs sounds in sheep with ovine pulmonary adenocarcinoma could be detected during auscultation between those recordings made at the margin of the tumour mass, directly over the tumour mass and in normal lung above the dorsal margin of the lesion [3]. Studies have highlighted the lack of correlation between lungs sounds and distribution of pathology in “wheel-barrow negative” cases of ovine pulmonary adenocarcinoma despite the OPA lesions extending to involve up to 20% of lung tissue [4].

*Trueperella pyogenes* is a common bacterial isolate from chronic suppurative pneumonia in cattle [15,20], and sheep [21]. Extended treatment with procaine penicillin in this study was effective where lung lesions did not extend dorsally more than 10 to 15 cm above the level on the chest wall indicated by the point of the olecranon however no long term survival data are available as culling was recommended because of the likelihood of recurrence following stress (e.g. next parturition). Many drugs are used to treat dairy cows when penicillin would have sufficed [22].

The good treatment response in 8 of 12 cows in the present study challenges the assertion [16,18] that all cattle with chronic suppurative pneumonia respond poorly to treatment, suffer chronic weight loss and may die as a result of an acute exacerbation of the condition. Furthermore, whether the dosing interval suits the current management [18] must not be considered an important factor in antibiotic selection. However, without accurate ultrasound assessment of the lungs [13], a provisional diagnosis of chronic suppurative pneumonia cannot be confirmed therefore treatment efficacy cannot be reliably evaluated.

Classification of the ultrasonographic changes into fine-, medium- and coarse-grained structures based upon the echogenic pattern with <10, 10–20 and >20 hyperechogenic zones per centimeter penetration of pneumonic lung tissue [13] was considered unnecessarily complicated and did not aid formulating the prognosis. Rather, the hypoechoic changes had a distinct columnar appearance dorsally representing the lobular distribution of superficial lung pathology extending to a large hypoechoic area with the sonographic appearance of liver most graphically illustrated when both pneumonic right ventral lung, diaphragm then liver were included in the same field Additional files 3, 4, 5, 6, 7, 8 and 9.

With some experience, ultrasound examination of both sides of the chest takes only five minutes and as such would not add significant cost to the veterinary examination in general practice. Moreover, establishing the correct diagnosis [13] and antibiotic therapy [22] may prove crucial to the likely recovery of the animal; six cattle in this study receiving treatment on referral improved after the antibiotic was changed to penicillin. Responsible antibiotic use by veterinarians necessitates an accurate diagnosis and ultrasound examination provides busy farm animal practitioners with a cost-effective ancillary test for chronic respiratory disease in adult cattle.

## Conclusions

Cattle with chronic suppurative pneumonia in this study often presented with a normal rectal temperature. Auscultation failed to identify the nature and extent of lung pathology in adult cattle with chronic suppurative pneumonia. Ultrasonographic examination of the chest provided critical information in the diagnosis of chronic suppurative pulmonary disease, and in formulating a prognosis. The next steps could include practical training of veterinary practitioners in ultrasonography so that this ancillary test can be more fully evaluated under field conditions [23]. Analysis of results generated by a large number of farm animal practitioners would allow prevalence data to be determined and treatment strategies to be evaluated with evidence-based recommendations to the benefit of our patients and farming clients. This regimen would go some way to meet the challenge that everyone involved in prescribing for cattle with respiratory disease should be striving to improve the accuracy of their diagnosis and treatment efficacy [18].

## Competing interests

The author declares that he has no competing interests.

## Supplementary Material

Additional file 1 **Heifer with chronic bronchopneumonia.** Recording over dorsal areas of right and left chest – normal lung sounds. Recordings over junction of normal lung dorsally and bronchopneumonia ventrally – there are no obvious adventitious sounds despite the extensive consolidation evident during the ultrasound examination. It is possible that the lung sounds resemble wheezes but they may also represent increased audibility of normal sounds exaggerated by the increased respiratory effort visible in the video recording? Recordings over bronchopneumonia ventrally – there are no obvious adventitious sounds despite the extensive consolidation evident during the ultrasound examination. The heart sounds are clearly audible. This heifer responded well to antibiotic therapy (Figures 1 and 15; videos 1 and 2) and was discharged 10 days after admission. Click here for file

Additional file 2 **Recording over dorsal area of the left chest – normal lung sounds.** Superimposed rumen sounds. Recordings over junction of normal lung dorsally and bronchopneumonia ventrally – there are no obvious adventitious sounds despite the extensive consolidation evident during the ultrasound examination. It is possible that the lung sounds resemble wheezes but they may also represent increased audibility of normal sounds exaggerated by the increased respiratory effort visible in the video recording? Recordings over bronchopneumonia ventrally – there are no obvious adventitious sounds despite the extensive consolidation evident during the ultrasound examination. Indeed, the lungs sounds are reduced in comparison with those recorded at the junction between normal lung and pathology (same volume recording setting). The heart sounds are not audible. This heifer failed to antibiotic therapy (video 5) and was euthanased for welfare reasons. Click here for file

Additional file 3 **Video recording 1.** The heifer calved unaided four weeks previously. Appetite is markedly reduced and the cow refuses the concentrate component of the ration. The flanks are very sunken suggesting a reduced appetite. Milk production is reduced to 50 per cent of expected yield (yielding 15 litres per day). The rectal temperature is 38.8°C. The cow is dull and depressed and often stands with the neck extended and the head held lowered. There is occasional coughing. A muco-purulent nasal discharge is present intermittently. The respiratory rate is increased to 48 breaths per minute with an abdominal effort. There are no other significant clinical findings. This heifer had been treated with marbofloxacin for five consecutive days without improvement before referral. As the probe head is advanced ventrally from normal lung tissue present in the dorsal lung field (bright hyperechoic [white] line moving in time with respiration), the first ultrasonographic change suggestive of bronchopneumonia is the columnar irregularity of the hyperechoic linear echo of the normal visceral (pulmonary) pleura in the antero-ventral apical and cardiac lung lobes. The dorsal margin of the lung pathology commenced 8–10 cm above the point of the elbow at the 6th and 7th intercostal spaces and extended from this level to the ventral margin of the lung lobes. These hypoechoic “columns” extended 2 to 8 cm from the visceral pleura and were bordered distally by bright hyperechoic lines as the sound waves contacted either normal aerated tissue or smaller airways deeper within the lung tissue. Ventrally, ultrasonographic examination imaged the diseased lung as a large hypoechoic area containing multiple 5–10 mm wide hyperechoic lines which extended up to 6–8 cm into the lung parenchyma. In was possible to image the right lung and liver in the same field; diseased lung had the sonographic density of liver. Click here for file

Additional file 4 **Video recording 2.** The same heifer as video 1 after 10 days’ treatment with procaine penicillin (s.i.d). Appetite is markedly improved and the cow is eating the full concentrate ration (6 kg/day). The flanks (rumen) are full suggesting a normal appetite. Milk production remains at 15 litres per day. The rectal temperature is 38.5°C. The cow is much brighter although there is still a mucoid nasal discharge. The respiratory rate is 30 breaths per minute with no abdominal effort. Click here for file

Additional file 5 **Video recording 3.** The cow calved unaided two months previously. Appetite is markedly reduced and the cow refuses the concentrate component of the ration. The flanks are very sunken suggesting a reduced appetite. Milk production has ceased. The rectal temperature is 38.5°C. The cow is dull and depressed and often stands with the neck extended and the head held lowered. There is occasional coughing. A muco-purulent nasal discharge is present intermittently. The respiratory rate is increased to 48 breaths per minute with an abdominal effort. There are severe injection site reactions in the left hip region where the cow has been injected by the farmer. As the probe head is advanced ventrally from normal lung tissue present in the dorsal lung field (bright hyperechoic [white] line moving in time with respiration), the first ultrasonographic change in lung parenchyma is the pronounced columnar irregularity of the hyperechoic linear echo of the normal visceral (pulmonary) pleura in the antero-ventral apical and cardiac lung lobes. The dorsal margin of the lung pathology commenced 10 cm above the point of the elbow at the 6th and 7th intercostal spaces and extended from this level to the ventral margin of the lung lobes. These hypoechoic “columns” extended up to 8 cm from the visceral pleura and were bordered distally by bright hyperechoic lines as the sound waves contacted either normal aerated tissue or smaller airways deeper within the lung tissue. Ventrally, ultrasonographic examination imaged the diseased lung as a large hypoechoic area containing multiple 5–20 mm wide hyperechoic lines which extended up to 8 cm into the lung parenchyma. Diseased lung had the sonographic density of liver. Please view video recording 4 to view the cow after treatment. Click here for file

Additional file 6 **Video recording 4.** The cow is shown much improved at discharge after 30 days’ penicillin therapy. There are no injection site swellings. The cow had been dried off because she was giving so little milk at admission. Click here for file

Additional file 7 **Video recording 5.** The heifer calved unaided four weeks previously. Appetite is markedly reduced and the cow refuses the concentrate component of the ration. The flanks are very sunken suggesting a reduced appetite. The heifer has been dried off due to the very low milk yield. The rectal temperature is 38.8°C. The heifer is dull and depressed and often stands with the neck extended and the head held lowered. There is occasional coughing. A purulent nasal discharge is present in both nostrils. The respiratory rate is increased to around 40 breaths per minute with an abdominal effort. There are no other significant clinical findings. As the probe head is advanced ventrally from normal lung tissue present in the dorsal lung field, the first ultrasonographic change in lung parenchyma is the pronounced columnar irregularity of the hyperechoic linear echo of the normal visceral (pulmonary) pleura in the antero-ventral apical and cardiac lung lobes. The dorsal margin of the lung pathology commences 15–20 cm above the point of the elbow at the 6th and 7th intercostal spaces and extends from this level to the ventral margin of the lung lobes. These hypoechoic “columns” extend up to 8 cm from the visceral pleura and are bordered distally by bright hyperechoic lines as the sound waves contact either normal aerated tissue or smaller airways deeper within the lung tissue. Ventrally, ultrasonographic examination images the diseased lung as a large hypoechoic area containing multiple 5–10 mm wide hyperechoic lines which extend up to 8 cm into the lung parenchyma. In is possible to image the right lung and liver in the same field; diseased lung has the sonographic density of liver. This heifer failed to respond to antibiotic therapy due to the extensive nature of the suppurative bronchopneumonia. Click here for file

Additional file 8 **Video recording 6.** This heifer failed to respond to antibiotic therapy due to the extensive nature of the suppurative bronchopneumonia. The dorsal margin of the lung pathology commences 15 cm above the point of the elbow at the 6th and 7th intercostal spaces and extends from this level to involve the ventral margin of the lung lobes. Ultrasonographic examination images the diseased lung as a large hypoechoic area containing multiple 5–10 mm wide hyperechoic lines which extend up to 8 cm into the lung parenchyma. There is an increased amount of pericardial fluid and adhesions between the pericardium and ventral lung. These changes are evident at necropy. *Necrospy findings.* The cadaver is placed in right lateral recumbency with the left lung presented. There is evidence of extensive suppurative bronchopneumonia with massive cellular infiltration and pus within the airways of the left apical and cardiac lung lobes (compare these changes with the sonographic results). There are adhesions between the pericardium and lung lobes, and an increased volume of pericardial fluid (transudate). Click here for file

Additional file 9 **Video recording 7.** The cow’s appetite is markedly reduced and the cow refuses the concentrate component of the ration. The flanks are very sunken suggesting a reduced appetite. Milk production has ceased. The rectal temperature is 38.5°C. The cow is dull and depressed and often stands with the neck extended and the head held lowered. The cow’s back is arched suggestive of thoracic/anterior abdominal pain. There is occasional coughing. A muco-purulent nasal discharge is present intermittently. The respiratory rate is increased to 60 breaths per minute with an abdominal effort. As the probe head is advanced ventrally from normal lung tissue present in the dorsal lung field the first ultrasonographic change in lung parenchyma is the pronounced columnar irregularity of the hyperechoic linear echo of the normal visceral (pulmonary) pleura in the antero-ventral apical and cardiac lung lobes. The dorsal margin of the lung pathology commenced 20 cm above the point of the elbow at the 6th and 7th intercostal spaces and extended from this level to the ventral margin of the lung lobes. These hypoechoic “columns” extended up to 8 cm from the visceral pleura and were bordered distally by bright hyperechoic lines as the sound waves contacted either normal aerated tissue or smaller airways deeper within the lung tissue. Diseased lung had the sonographic density of liver. This cow failed to respond to antibiotic therapy due to the extensive nature of the lesions. Click here for file
