# Erratum to: ‘Clinical presentation, auscultation recordings, ultrasonographic findings and treatment response of 12 adult cattle with chronic suppurative pneumonia: case study’

**DOI:** 10.1186/s13620-016-0064-7

**Published:** 2016-04-27

**Authors:** Philip R. Scott

**Affiliations:** Easter Bush Veterinary Centre, Roslin, Midlothian, EH25 9RG, Scotland UK

Unfortunately, the original version of this article [1] contained errors. The additional files were displayed in the wrong order and also duplicated where video 6 was identical to video 4 and video 7 was identical to video 5. The video files have been included here in the correct sequence.

Additional files 3, 4, 5, 6, 7, 8 and 9 have been included to further illustrate adult cattle with chronic suppurative pneumonia. Each video file has its own caption which gives more detail.

## Additional files

Additional file 3: Video recording 1. The heifer calved unaided four weeks previously. Appetite is markedly reduced and the cow refuses the concentrate component of the ration. The flanks are very sunken suggesting a reduced appetite. Milk production is reduced to 50 per cent of expected yield (yielding 15 litres per day). The rectal temperature is 38.8 °C. The cow is dull and depressed and often stands with the neck extended and the head held lowered. There is occasional coughing. A muco-purulent nasal discharge is present intermittently. The respiratory rate is increased to 48 breaths per minute with an abdominal effort. There are no other significant clinical findings. This heifer had been treated with marbofloxacin for five consecutive days without improvement before referral. As the probe head is advanced ventrally from normal lung tissue present in the dorsal lung field (bright hyperechoic [white] line moving in time with respiration), the first ultrasonographic change suggestive of bronchopneumonia is the columnar irregularity of the hyperechoic linear echo of the normal visceral (pulmonary) pleura in the antero-ventral apical and cardiac lung lobes. The dorsal margin of the lung pathology commenced 8–10 cm above the point of the elbow at the 6th and 7th intercostal spaces and extended from this level to the ventral margin of the lung lobes. These hypoechoic “columns” extended 2 to 8 cm from the visceral pleura and were bordered distally by bright hyperechoic lines as the sound waves contacted either normal aerated tissue or smaller airways deeper within the lung tissue. Ventrally, ultrasonographic examination imaged the diseased lung as a large hypoechoic area containing multiple 5–10 mm wide hyperechoic lines which extended up to 6–8 cm into the lung parenchyma. In was possible to image the right lung and liver in the same field; diseased lung had the sonographic density of liver. (MOV 19 MB) (MOV 19186 kb)

Additional file 4: Video recording 2. The same heifer as video 1 after 10 days’ treatment with procaine penicillin (s.i.d). Appetite is markedly improved and the cow is eating the full concentrate ration (6 kg/day). The flanks (rumen) are full suggesting a normal appetite. Milk production remains at 15 litres per day. The rectal temperature is 38.5 °C. The cow is much brighter although there is still a mucoid nasal discharge. The respiratory rate is 30 breaths per minute with no abdominal effort. (MOV 5 MB) (MOV 5032 kb)

Additional file 5: Video recording 3. The cow calved unaided two months previously. Appetite is markedly reduced and the cow refuses the concentrate component of the ration. The flanks are very sunken suggesting a reduced appetite. Milk production has ceased. The rectal temperature is 38.5 °C. The cow is dull and depressed and often stands with the neck extended and the head held lowered. There is occasional coughing. A muco-purulent nasal discharge is present intermittently. The respiratory rate is increased to 48 breaths per minute with an abdominal effort. There are severe injection site reactions in the left hip region where the cow has been injected by the farmer. As the probe head is advanced ventrally from normal lung tissue present in the dorsal lung field (bright hyperechoic [white] line moving in time with respiration), the first ultrasonographic change in lung parenchyma is the pronounced columnar irregularity of the hyperechoic linear echo of the normal visceral (pulmonary) pleura in the antero-ventral apical and cardiac lung lobes. The dorsal margin of the lung pathology commenced 10 cm above the point of the elbow at the 6th and 7th intercostal spaces and extended from this level to the ventral margin of the lung lobes. These hypoechoic “columns” extended up to 8 cm from the visceral pleura and were bordered distally by bright hyperechoic lines as the sound waves contacted either normal aerated tissue or smaller airways deeper within the lung tissue. Ventrally, ultrasonographic examination imaged the diseased lung as a large hypoechoic area containing multiple 5–20 mm wide hyperechoic lines which extended up to 8 cm into the lung parenchyma. Diseased lung had the sonographic density of liver. Please view video recording 4 to view the cow after treatment. (MOV 18989 kb)

Additional file 6: Video recording 4. The cow is shown much improved at discharge after 30 days’ penicillin therapy. There are no injection site swellings. The cow had been dried off because she was giving so little milk at admission. (MOV 3013 kb)

Additional file 7: Video recording 5. The heifer calved unaided four weeks previously. Appetite is markedly reduced and the cow refuses the concentrate component of the ration. The flanks are very sunken suggesting a reduced appetite. The heifer has been dried off due to the very low milk yield. The rectal temperature is 38.8 °C. The heifer is dull and depressed and often stands with the neck extended and the head held lowered. There is occasional coughing. A purulent nasal discharge is present in both nostrils. The respiratory rate is increased to around 40 breaths per minute with an abdominal effort. There are no other significant clinical findings. As the probe head is advanced ventrally from normal lung tissue present in the dorsal lung field, the first ultrasonographic change in lung parenchyma is the pronounced columnar irregularity of the hyperechoic linear echo of the normal visceral (pulmonary) pleura in the antero-ventral apical and cardiac lung lobes. The dorsal margin of the lung pathology commences 15–20 cm above the point of the elbow at the 6th and 7th intercostal spaces and extends from this level to the ventral margin of the lung lobes. These hypoechoic “columns” extend up to 8 cm from the visceral pleura and are bordered distally by bright hyperechoic lines as the sound waves contact either normal aerated tissue or smaller airways deeper within the lung tissue. Ventrally, ultrasonographic examination images the diseased lung as a large hypoechoic area containing multiple 5–10 mm wide hyperechoic lines which extend up to 8 cm into the lung parenchyma. In is possible to image the right lung and liver in the same field; diseased lung has the sonographic density of liver. This heifer failed to respond to antibiotic therapy due to the extensive nature of the suppurative bronchopneumonia. (MOV 16056 kb)

Additional file 8: Video recording 6. This heifer failed to respond to antibiotic therapy due to the extensive nature of the suppurative bronchopneumonia. The dorsal margin of the lung pathology commences 15 cm above the point of the elbow at the 6th and 7th intercostal spaces and extends from this level to involve the ventral margin of the lung lobes. Ultrasonographic examination images the diseased lung as a large hypoechoic area containing multiple 5–10 mm wide hyperechoic lines which extend up to 8 cm into the lung parenchyma. There is an increased amount of pericardial fluid and adhesions between the pericardium and ventral lung. These changes are evident at necropy. *Necrospy findings*. The cadaver is placed in right lateral recumbency with the left lung presented. There is evidence of extensive suppurative bronchopneumonia with massive cellular infiltration and pus within the airways of the left apical and cardiac lung lobes (compare these changes with the sonographic results). There are adhesions between the pericardium and lung lobes, and an increased volume of pericardial fluid (transudate). (MOV 12058 kb)

Additional file 9: Video recording 7. The cow’s appetite is markedly reduced and the cow refuses the concentrate component of the ration. The flanks are very sunken suggesting a reduced appetite. Milk production has ceased. The rectal temperature is 38.5 °C. The cow is dull and depressed and often stands with the neck extended and the head held lowered. The cow’s back is arched suggestive of thoracic/anterior abdominal pain. There is occasional coughing. A muco-purulent nasal discharge is present intermittently. The respiratory rate is increased to 60 breaths per minute with an abdominal effort. As the probe head is advanced ventrally from normal lung tissue present in the dorsal lung field the first ultrasonographic change in lung parenchyma is the pronounced columnar irregularity of the hyperechoic linear echo of the normal visceral (pulmonary) pleura in the antero-ventral apical and cardiac lung lobes. The dorsal margin of the lung pathology commenced 20 cm above the point of the elbow at the 6th and 7th intercostal spaces and extended from this level to the ventral margin of the lung lobes. These hypoechoic “columns” extended up to 8 cm from the visceral pleura and were bordered distally by bright hyperechoic lines as the sound waves contacted either normal aerated tissue or smaller airways deeper within the lung tissue. Diseased lung had the sonographic density of liver. This cow failed to respond to antibiotic therapy due to the extensive nature of the lesions. (MOV 15489 kb)
